# Supplementary material for: Stress across the Lifespan: From Risk to Management—Conference Report on the Inaugural Canadian Stress Research Summit
Source: Int J Environ Res Public Health. 2022 Sep 3;19(17):11015. doi: 10.3390/ijerph191711015 (PMC9517807; doi:10.3390/ijerph191711015)

International Journal of
Environmental Research and Public Health

MDPI

Supplementary Materials

All scientific abstracts may be reviewed here:

https://www.torontomu.ca/content/dam/canadian-srs/CSRS_2021_Abstract_Book.pdf

Supplemental Figures

Supplemental Figure S1. View of Gather (a) Poster Room and (b) Roof Top Lounge.

a.

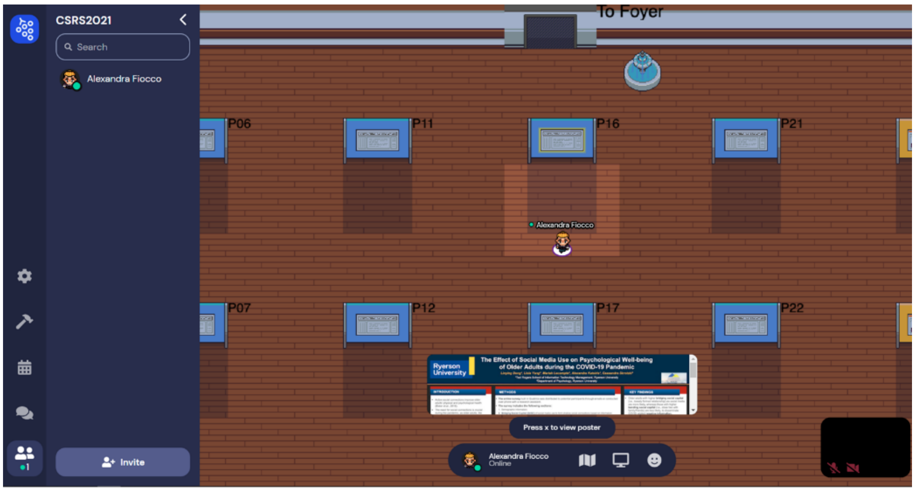

b.

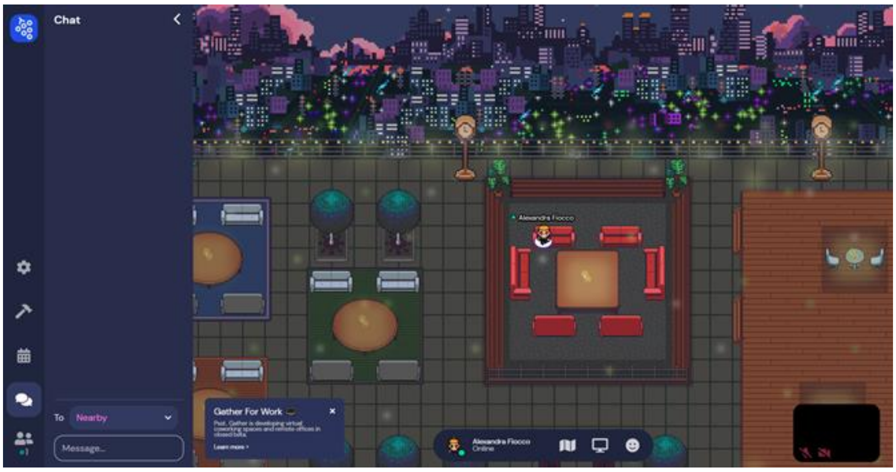

Supplemental Figure S2. Graphical Synthesis of Dr. Lupien's Keynote: *From Neurotoxicity to Vulnerability: A Developmental Perspective of the Effect of Stress on the Brain.*

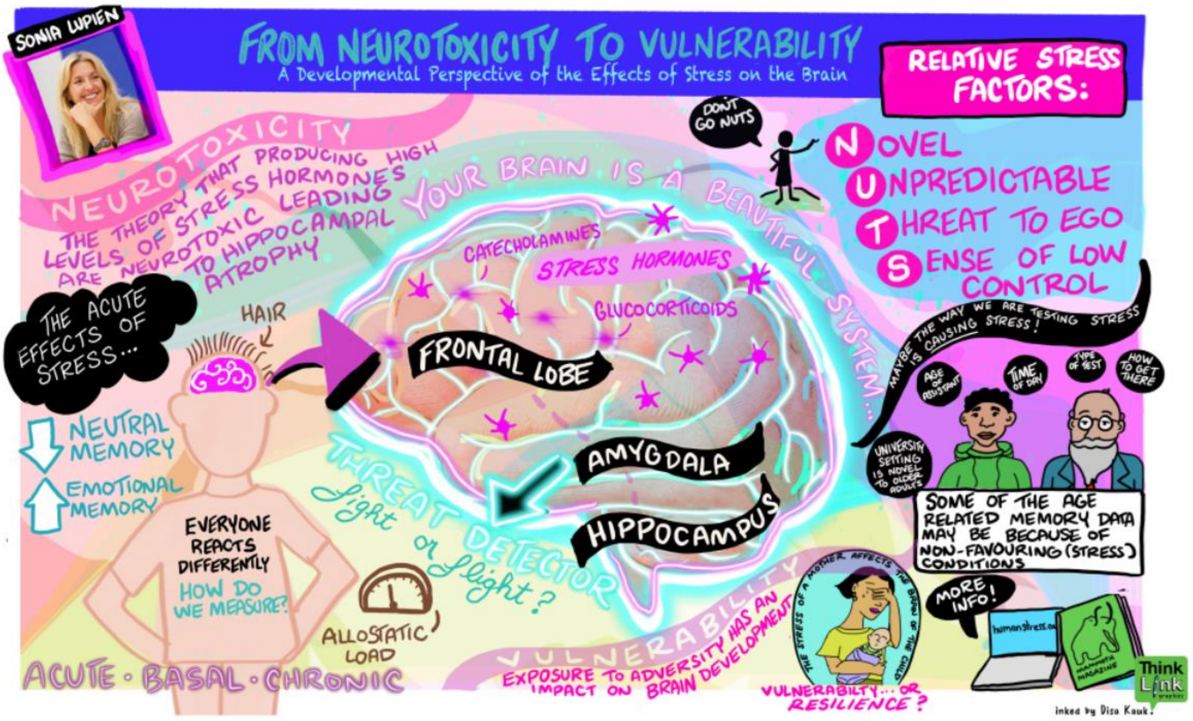

Supplemental Figure S3. Graphical Synthesis of Dr. Matthew Hill's Keynote, entitled *A Tale of Translation: Endocannabinoid Regulation of Stress, Anxiety and Fear, From Rodent to Humans.*

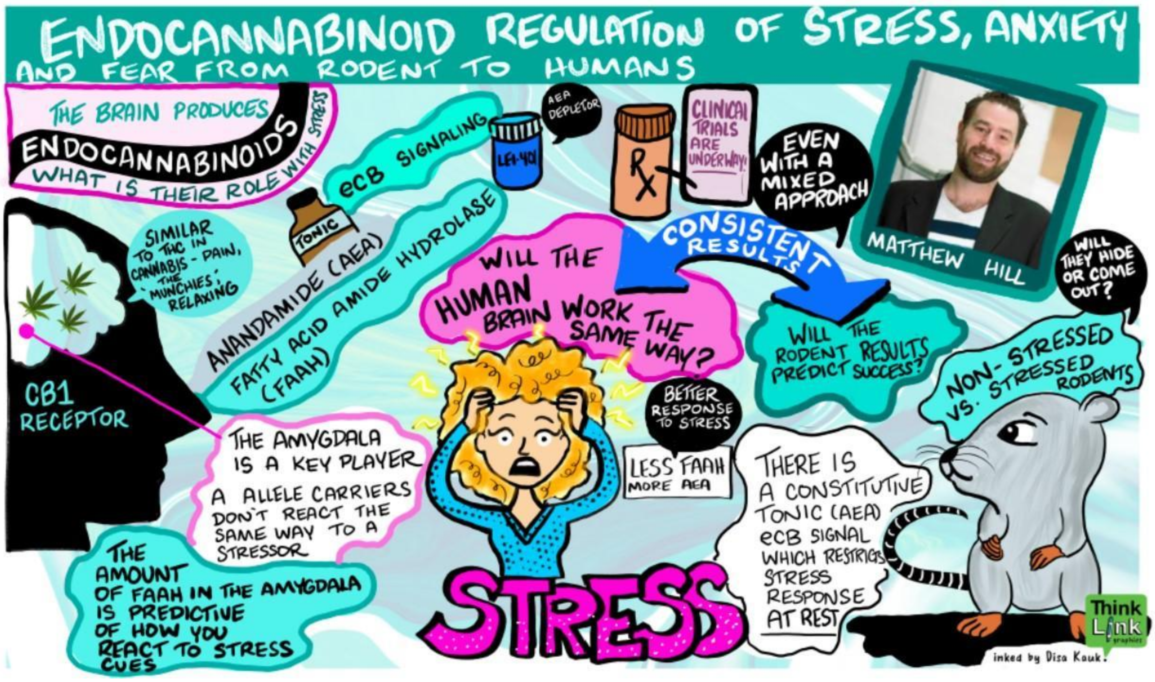

Supplemental Figure S4. Graphical Synthesis of Stress and Indigenous Health: Teachings of Blood Memory and Epigenetics.

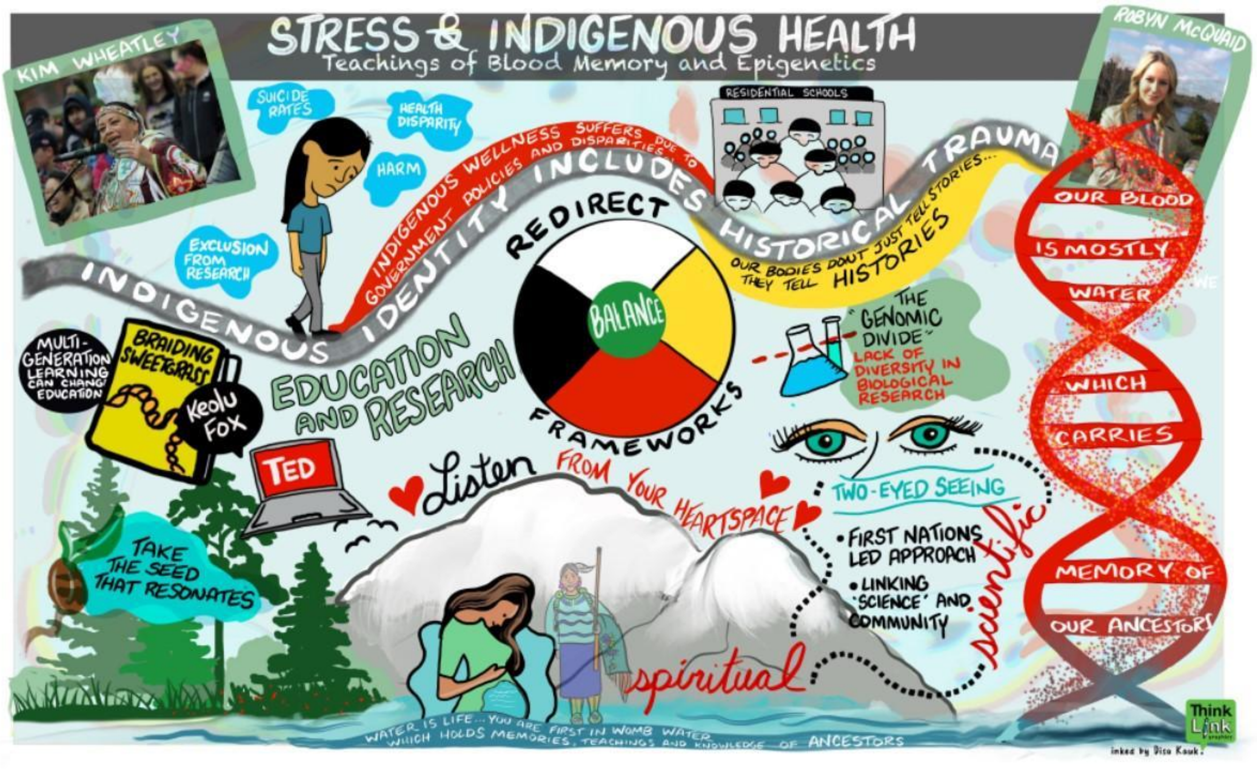

Supplemental Figure S5. Graphical Synthesis of the *COVID-19 Pandemic* Symposium.

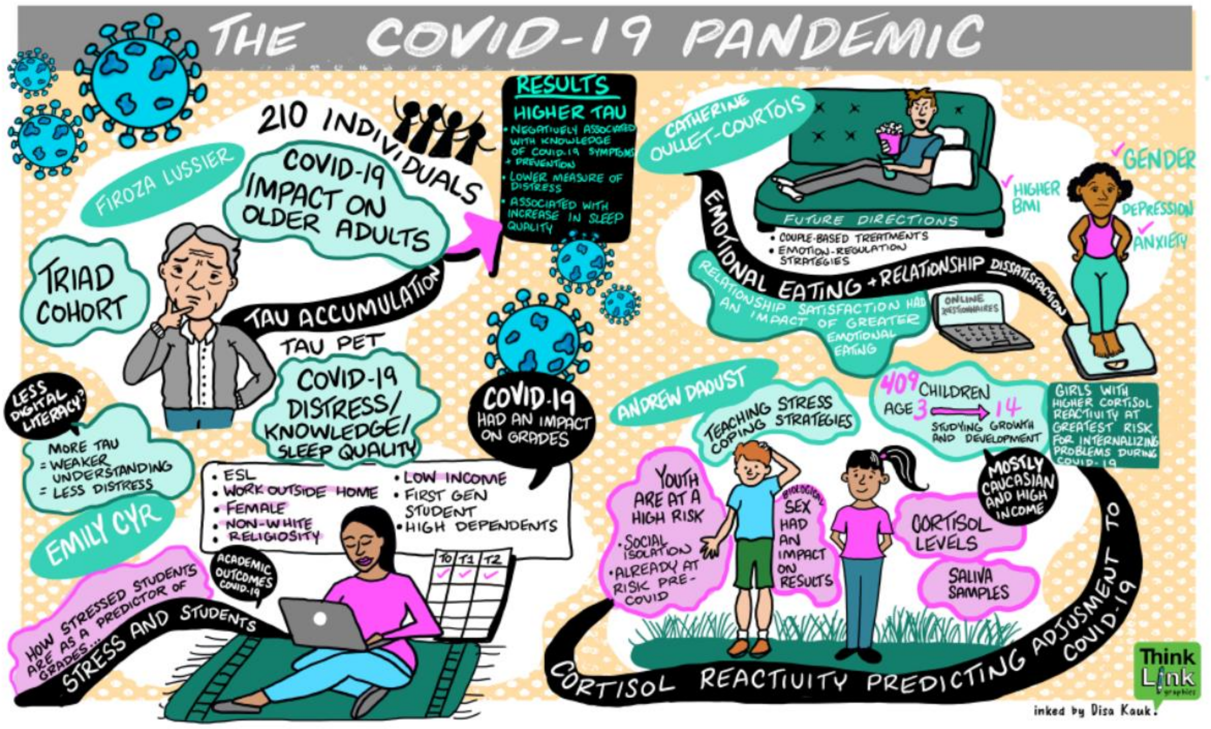

Supplemental Figure S6. Graphics Synthesis of Dr. Nasreen Khati’s public presentation *Mental Health in 2021: The Echo Pandemic*.

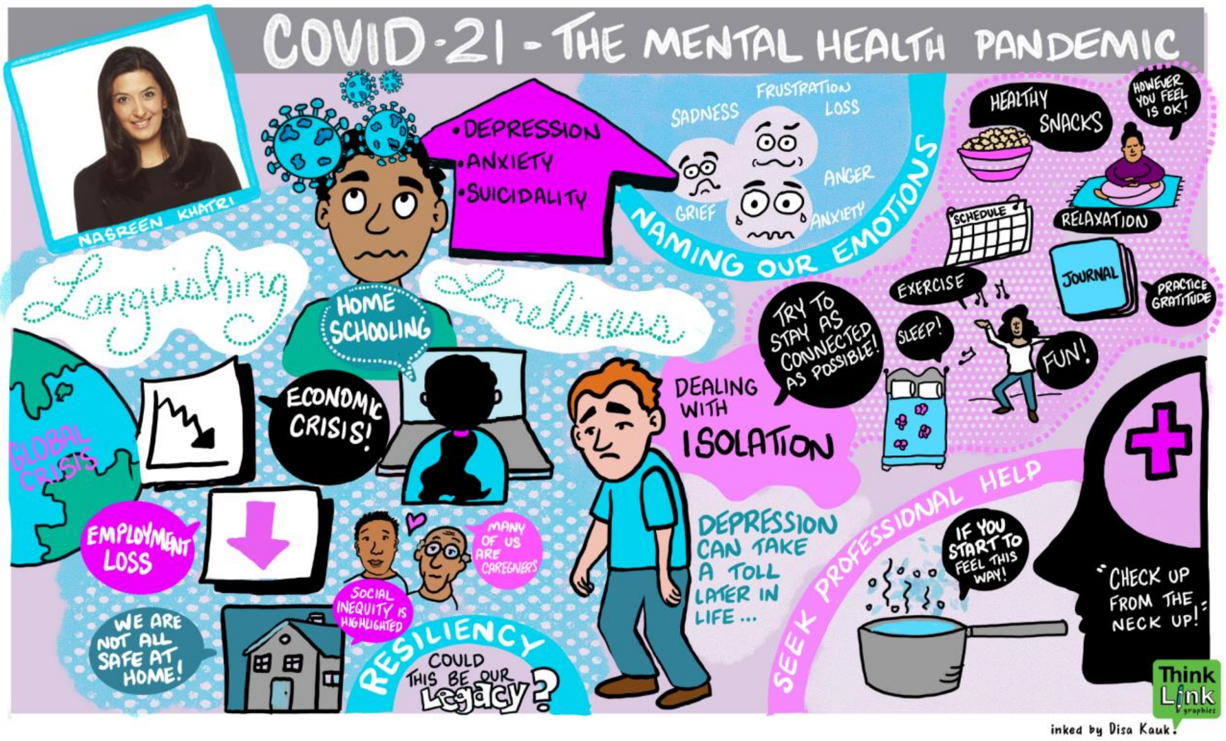

Supplement: Supplementary file 1 [file ijerph-19-11015-s001.zip › ijerph-1830795-supplementary.pdf]
